# Blue-light stimulation of the blind-spot constricts the pupil and enhances contrast sensitivity

**DOI:** 10.1371/journal.pone.0286503

**Published:** 2023-05-31

**Authors:** Tim Schilling, Mojtaba Soltanlou, Hans-Christoph Nuerk, Hamed Bahmani

**Affiliations:** 1 Dopavision GmbH, Berlin, Germany; 2 Department of Psychology, University of Tuebingen, Tuebingen, Germany; 3 School of Psychology, University of Surrey, Guildford, United Kingdom; 4 LEAD Graduate School & Research Network, University of Tuebingen, Tuebingen, Germany; 5 Bernstein Center for Computational Neuroscience, Tuebingen, Germany; Technical University of Munich, Germany, UNITED KINGDOM

## Abstract

Short- and long-wavelength light can alter pupillary responses differently, allowing inferences to be made about the contribution of different photoreceptors on pupillary constriction. In addition to classical retinal photoreceptors, the pupillary light response is formed by the activity of melanopsin-expressing intrinsically photosensitive retinal ganglion cells (ipRGC). It has been shown in rodents that melanopsin is expressed in the axons of ipRGCs that bundle at the optic nerve head, which forms the perceptual blind-spot. Hence, the first aim of this study was to investigate if blind-spot stimulation induces a pupillary response. The second aim was to investigate the effect of blind-spot stimulation by using the contrast sensitivity tests. Fifteen individuals participated in the pupil response experiment and thirty-two individuals in the contrast sensitivity experiment. The pupillary change was quantified using the post-illumination pupil response (PIPR) amplitudes after blue-light (experimental condition) and red-light (control condition) pulses in the time window between 2 s and 6 s post-illumination. The contrast sensitivity was assessed using two different tests: the Freiburg Visual Acuity Test and Contrast Test and the Tuebingen Contrast Sensitivity Test, respectively. Contrast sensitivity was measured before and 20 minutes after binocular blue-light stimulation of the blind-spot at spatial frequencies higher than or equal to 3 cycles per degree (cpd) and at spatial frequencies lower than 3 cpd (control condition). Blue-light blind-spot stimulation induced a significantly larger PIPR compared to red-light, confirming a melanopsin-mediated pupil-response in the blind-spot. Furthermore, contrast sensitivity was increased after blind-spot stimulation, confirmed by both contrast sensitivity tests. Only spatial frequencies of at least 3 cpd were enhanced. This study demonstrates that stimulating the blind-spot with blue-light constricts the pupil and increases the contrast sensitivity at higher spatial frequencies.

## Introduction

A characteristic feature of the retina is the optic disc, where the axons of retinal ganglion cells bundle to form a head of the optic nerve. No classical photoreceptor cells–cones and rods–are located inside the optic disc, hence the light hitting it directly is invisible to the observer [1,2]. For this reason, the optic disc is also known as the blind-spot. The optic disc contains the axons of intrinsically photosensitive retinal ganglion cells (ipRGCs) [3]. ipRGCs express the photopigment melanopsin which is maximally excited by blue-light (λ_peak_ = 480 nm) in the short-wavelength range of the visible spectrum, but not long-wavelength red-light [4]. It has been shown in rodents and primates that melanopsin is present in the axons of ipRGCs as well [5,6]. Hence the stimulation of the optic disc has a potential to evoke electrophysiological responses from the inner plexiform layer and from the retinal ganglion cells [7,8].

The ipRGCs role in the pupillary light response has been well established [9]. Specifically, they contribute to the slow recovery component—an extended response mediated primarily by melanopsin activation that persists for some time after cessation of the light stimulus–the so-called Post-Illumination Pupillary Response (PIPR) [10]. Supporting evidence exists that melanopsin drives PIPR, which is a sustained pupillary constriction after stimulus deactivation and is generated by the intrinsic response of ipRGCs [6,11,12]. Whereas both rods and ipRGCs have been shown to provide input to the pupillary light response, and thus rhodopsin and melanopsin contribute largely to PIPR <1.7 s, melanopsin has been reported to dominate all phases of PIPR and contribute exclusively to PIPR after 1.7 s [11–13].

This study was conducted to investigate the contrast sensitivity of the eye after blue-light stimulation of the blind-spot. For the binocular blind-spot stimulation, a non-invasive stimulation technique using a virtual reality headset was developed [7,14]. Firstly, we confirmed that the blind-spot is more sensitive to blue-light in comparison with red-light using the pupil response assay by testing the hypothesis that blue-light (experimental condition) stimulation of the blind-spot shows a larger pupillary change (constriction) as compared to red-light (control condition). Secondly, we continued by assessing the contrast sensitivity before and after blind-spot stimulation with blue-light.

Various non-invasive clinical contrast sensitivity tests are available. We first assessed contrast sensitivity using the Freiburg Visual Acuity and Contrast Test (FrACT, Version 3.9.8), which is available online and can be performed on regular monitors [15,16] (experiment 2.1). In the second contrast sensitivity experiment, we used the Tuebingen Contrast Sensitivity Test (TueCST), which has a higher gray-scale resolution, high repeatability, and excellent reliability [17] (experiment 2.2). As such, the effect was expected to be more pronounced in experiment 2.2 compared to experiment 2.1. We tested the hypothesis that stimulating the blind-spot with blue-light increases contrast sensitivity at spatial frequencies higher than or equal to 3 cycles per degree (cpd). We expected no stimulation effect at spatial frequencies lower than 3 cpd (control conditions) and thus we also tested separately the hypothesis for lower than 3 cpd. Frequencies above 3 cpd were classified as experimental conditions because an effect in this frequency range was expected, following previous work by Domenici et al. [18]. The control condition with frequencies lower than 3 cpd was used to show the specificity of stimulation in the experimental condition.

## Materials and methods

### Participants

A cumulative total of 47 participants were recruited for this study (22 female and 25 male; age range from 20 to 38 years (mean ± standard deviation was 27.5 ± 4.5 years). For the first experiment (pupil light response) 15 participants (experiment 1), and for the second experiments (contrast sensitivity) 22 participants for FrACT (experiment 2.1) and 10 participants for TueCST (experiment 2.2) were recruited. The participants who took part in the study had, following the inclusion criteria, normal or corrected-to-normal vision (prescription spectacles or contact lenses). After explaining the experiment, written informed consent was obtained from all participants. The following exclusion criteria were used: eye disease, more than -3 D of spherical error, more than -2 D of astigmatism, participation in other clinical examinations, and known structural and/or functional anomalies of the eye apparatus (e.g., cataract, glaucoma, strabismus, keratoconus, anisocoria). No participants were excluded. Visual acuity was assessed binocularly before the experiment with FrACT [15,16] to be greater or equal to 0.0 logMAR. Both experiments were approved by the Ethics Committee of the Department of Psychology of the University of Tuebingen and conducted in accordance with the tenets of the Declaration of Helsinki.

### Light stimulus protocol

#### Experiment 1: Pupil light response

**Fig 1:**
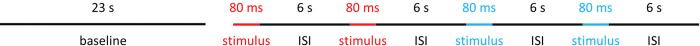
Light stimulus protocol for the pupil light response experiment.

The pupil light response experiment was performed as described previously [19], with a monitor placed 50 cm in front of the chin rest where the participant has stabilized the head. Briefly, after 23 s of baseline (no blind-spot stimulus), where the participant fixated in the middle of the four fixation targets, two red and two blue stimuli of 80 ms each were followed before the recording of PIPR during an inter-stimulus-interval (ISI) that lasted 6 s, see Fig 1.

**Fig 2:**
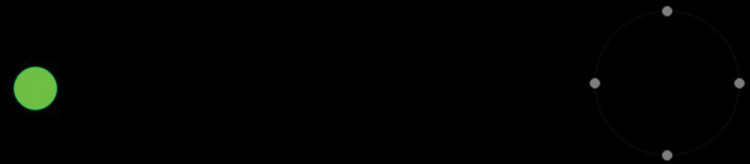
Screenshot of blind-spot mapping calibration. Left: Green stimulus, right: Four fixation targets.

Pupil size and gaze position of the left eye were recorded using an eye tracker (EyeLink 1000 eye tracking system, SR Research Ltd., Ottawa, Ontario, Canada), while the right eye was covered with an eye patch. The blind-spot mapping was controlled by four fixation targets. While looking at the fixation target, the participant could use a keyboard to set the position and radius of the stimulus disc appearing on the screen. The stimulus size and the position had to be adjusted by the participant until the stimulus was not visible to the participant when fixating on all four surrounding fixation points as well as the centered fixation target, see Fig 2. The participants were instructed to look at the centered fixation target where the blind-spot stimulus would be completely invisible. To ensure that the fixation was within the fixation targets, the gaze position was monitored. During blind-spot stimulation, the gaze positions were 97.5 ± 1% within the fixation targets. Furthermore, the blind-spot stimulus diameter was much smaller with 1.25° than an average blind-spot diameter 5.5° horizontally and 7.7° vertically [20]. More details of experiment 1 have already been published in a conference proceeding paper [19]. Additionally, the spectral information of the monitor (FUJITSU Display B24-8 TS), which was used for blue and red blind-spot stimuli and was measured with i1 studio (x-rite Incorporated, Kentwood, Michigan, USA) that was placed on the screen of the monitor, and the software f.luxometer™ LLC (Los Angeles, California, USA), has been added under S1 Data. Furthermore, the experiment 1 was conducted in a darkened room.

#### Experiment 2: Contrast sensitivity tests

In experiment 2, the stimulation of the blind-spot was delivered by a custom-developed software through a smartphone, Android-based smartphone Samsung Galaxy S7 (Samsung, Seoul, South Korea) with a Super AMOLED display (5.1 inches, 1440x2560 pixels). The smartphone was placed in Exos 2 Virtual Reality Glasses for smartphone (Trust International B.V., Dordrecht, Netherlands), which is referred here as a virtual reality (VR) system. Before and 20 min after stimulation of the blind-spot binocularly with pulses of blue-light flickering at 15 Hz for 1 min, contrast sensitivity for 1, 3, 6 and 12 cpd was measured with the FrACT, and for 0.5, 3, 6 and 9 cpd with the TueCST. Each spatial frequency was assessed with 50 trials (repeated tasks to press a button after visual input), as with this trial number the TueCST and FrACT showed good repeatability and excellent reliability [17]. Before the experiment participants were trained with 10 trials for each spatial frequency. Feedback via beep sound was provided for correct and incorrect responses. The stimuli for contrast sensitivity assessment were Gabor Patch stimuli. These Gabor Patch stimuli had a stimulus size of 1.7° and were presented for 300 ms to avoid contrast adaptation. The screen to display the contrast sensitivity test used a luminance of 1.8 cd/m^2^ for FrACT on the XPS 13 9360 monitor (Dell GmbH, Frankfurt am Main, Germany), and 40 cd/m^2^ for TueCST [17] on a ViewPixx monitor (VPixx Technologies Inc., Saint-Bruno, Canada). The distance from the chin rest to the screen was 50 cm. In all experiments, the room was darkened to exclude sunlight. In the contrast sensitivity experiment, the ceiling illumination was kept constant throughout the experimental period. The intensity of the blue blind-spot stimulus was above the threshold of melanopsin by more than 13.8 log quanta.cm^-2^.s^-1^ (6.2 x 10^13^ photons/cm^2^) [21,22]. The spectral light information of the Samsung Galaxy S7, measured with i1 studio (x-rite Incorporated, Kentwood, Michigan, USA) and the software f.luxometer™ LLC (Los Angeles, California, USA), can be found under S2 Data.

**Fig 3:**
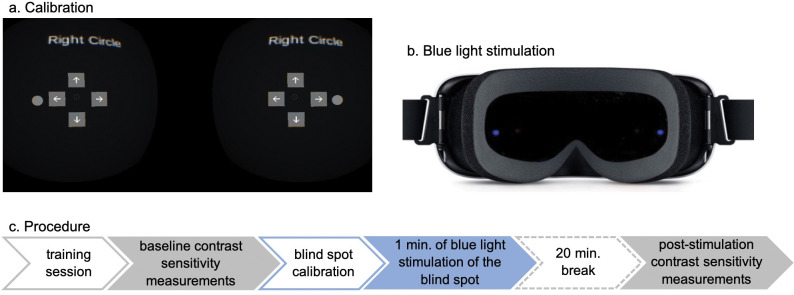
An overview of the virtual reality calibration and stimulation processes, and an outline of the overall procedure. The blind-spot calibration procedure (a), and a schematic illustration of the blue-light stimulation of the blind-spot in the virtual reality system (b). The outline of a protocol that participants underwent (c).

In order to allow participants to adjust the stimulus to their unique blind-spot position, the following calibration procedure was used. First, the participant placed the VR system on their head. Next, the participant selected either the left or the right stimulus circle. While looking at the central fixation cross, the participant could adjust the position of the stimulus using the arrow keys (Fig 3A) until it was no longer visible. Participants could select the arrow keys by moving their heads until the fixation target landed on one of the arrow keys, which caused the stimulus to move. In between head movements, the participant could repeatedly check whether the stimulus was on their blind-spot. The fixation target was a thin gray annulus with a dot in the center. While calibration occurred with a gray stimulus, a blue stimulus was used to stimulate the blind-spot for 1 min, see Fig 3B.

#### Statistical analysis

In experiment 1, one-tailed paired t-tests were performed between blue-light (experimental condition) and red-light (control condition) for four different time windows (between 2 s and 6 s, at 6 s, >1.8 s, and <1.7 s post-illumination). For the analysis of pupillary changes, the standards of pupillography were followed [23]. In experiment 2, two repeated measure ANOVAs were conducted for the two tests with the within-subject factors of 2 (time: pre-test and post-test) by 3 (spatial frequency: 3, 6, 12 cpd for FrACT, and 3, 6, 9 cpd for TueCST). To disentangle any observed effects, we followed up using Tukey post hoc tests. To check for the specificity of the blue-light stimulation on spatial frequencies higher than or equal to 3 cpd, we tested spatial frequencies lower than 3 cpd as well (control conditions). We conducted a paired *t*-test for 1 cpd for FrACT to compare pre-test and post-test, and a separate repeated measure ANOVA with the within-subject factors of 2 (time: pre-test and post-test) by 2 (spatial frequency: 0.5 and 1 cpd for TueCST). For statistical analysis jamovi (1.1.9.0, The jamovi project, 2020) was used.

Cohen’s d effect sizes are categorized as small (0.2), medium (0.5), and large (0.8). This means that the difference between the means of two conditions are less than 0.2, 0.5 or 0.8 of the standard deviations. Partial eta-squared is a measure of the effect size that shows the proportion of variance explained by a given variable in the model. It indicates the percentage of the variance in the dependent variable that attributes to the independent variable. Partial eta squared shows an effect size that explains how large the effect of an independent variable on the dependent variable is [24].

## Results

### Experiment 1: Pupil light response

**Fig 4:**
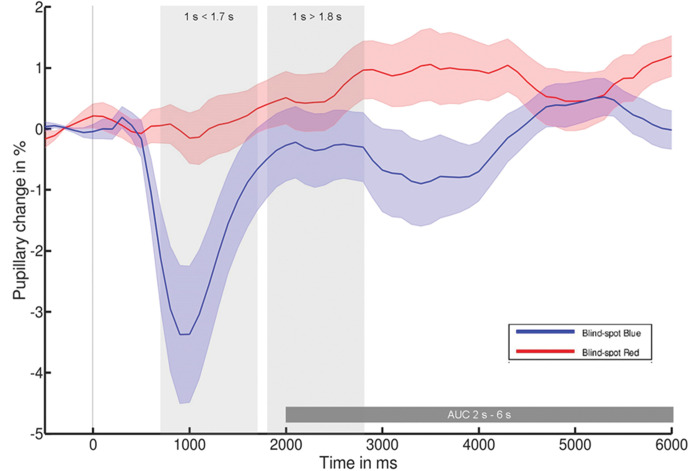
Pupillary change following blue- and red-light stimulation of the blind-spot (reprinted and modified from Fig 3 in [19], distributed under the terms of the Creative Commons Attribution License (CC BY-NC-ND 4.0)).

We first tested the pupillary light response change to blue- and red-light stimulus, respectively. A one-tailed paired t-test comparison was performed and showed a significantly larger pupillary change (constriction) to blue-light (experimental condition) as compared to red-light (control condition) between 2 s and 6 s (*t*(14) = -1.9, *p* = 0.043, *d* = -0.48) and at 6 s post-illumination (*t*(14) = -2.07, *p* = 0.029, *d* = -0.54). No significant difference was found for >1.8 s between blue- and red-light stimulus (*t*(14) = -1.2, *p* = 0.125, *d* = -0.31), but highly significant difference in pupillary change to blue- compared to red-light for <1.7 s (*t*(14) = -2.71, *p* = 0.009, *d* = -0.70), see Fig 4.

### Experiment 2.1: Freiburg contrast test

**Fig 5:**
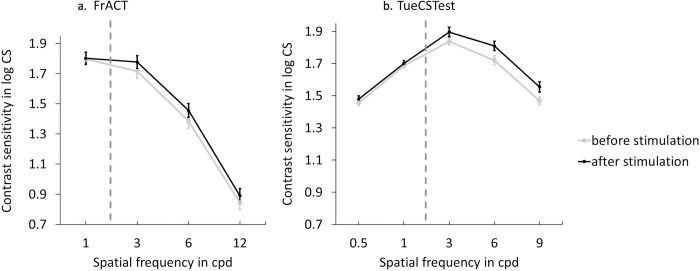
Mean and standard error of the mean of contrast sensitivity function in log CS before and after blue-light stimulation using FrACT (a) or TueCST (b) test, respectively. Dotted line indicates separation between lower than 3 cpd (control conditions) and higher than or equal to 3 cpd (experimental conditions). In TueCST experiment n = 10, in FrACT experiment n = 22.

The repeated measure ANOVA revealed a significant main effect of time (*F*(1, 9) = 5.85, *p* = 0.025, *η*_p_^2^ = 0.218) showing that contrast sensitivity was significantly lower before blue-light stimulation than 20 min after blue-light stimulation. A significant main effect of spatial frequency (*F*(2, 42) = 204.40, *p* < 0.001, *η*_p_^2^ = 0.907) was also observed for spatial frequencies of 3, 6, and 12 cpd. Further Tukey’s corrected post hoc paired *t*-tests for factor spatial frequency returned that contrast sensitivity significantly decreased from 3 cpd to 6 cpd, from 3 cpd to 12 cpd, and from 6 cpd to 12 cpd (*t*(42) = 7.41, *p* < 0.001; *t*(42) = 20.00, *p* < 0.001; *t*(42) = 12.59, *p* < 0.001, respectively) (Fig 5A). There was no significant interaction of time and spatial frequency (*F*(2, 42) = 0.21, *p* = 0.816, *η*_p_^2^ = 0.01).

For the control spatial frequencies, a paired *t*-test showed no significant change between before and 20 min after blue-light stimulation in the 1 cpd condition (*t*(21) = 0.06, *p* = 0.950, *d* = 0.012), showing that contrast sensitivity was not influenced by blue-light stimulation for spatial frequencies lower than 3 cpd.

### Experiment 2.2: Tuebingen contrast sensitivity test

The repeated measure ANOVA revealed a significant main effect of time (*F*(1, 9) = 6.95, *p* = 0.027, *η*_p_^^2^^ = 0.436) showing that contrast sensitivity was significantly lower before blue-light stimulation than 20 min after blue-light stimulation. A significant main effect of the factor spatial frequency (*F*(2, 18) = 128.94, *p* < 0.001, *η*_p_^^2^^ = 0.935) was also observed for spatial frequencies of 3, 6, and 9 cpd. Tukey’s corrected post hoc paired *t*-test for factor spatial frequency showed that contrast sensitivity significantly decreased from 3 cpd to 6 cpd, from 3 cpd to 9 cpd, and from 6 cpd to 9 cpd (*t*(18) = 4.48, *p* < 0.001; *t*(18) = 15.60, *p* < 0.001; *t*(18) = 11.11, *p* < 0.001, respectively) (Fig 5B). There was no significant interaction between time and spatial frequency (*F*(2, 18) = 0.18, *p* = 0.839, *η*_p_^^2^^ = 0.019).

For the control spatial frequencies, a separate repeated measure ANOVA on 0.5 cpd and 1 cpd revealed no significant effect of time (*F*(1, 9) = 0.94, *p* = 0.36, *η*_p_^^2^^ = 0.09) showing that contrast sensitivity was not influenced by blue-light stimulation for spatial frequencies lower than 3 cpd. A significant main effect of spatial frequency (*F*(1, 9) = 128.55, *p* < 0.001, *η*_p_^^2^^ = 0.94) but no interaction (*F*(1, 9) = 0.11, *p* = 0.75, *η*_p_^^2^^ = 0.12) were observed. Tukey’s corrected post hoc paired *t*-test for the factor spatial frequency showed that contrast sensitivity significantly increased from 0.5 cpd to 1 cpd (*t*(9) > 11.30, *p* < 0.001).

## Discussion

Blind-spot stimulation with blue-light induced a significantly larger pupil light response compared to red-light. Furthermore, blue-light stimulation increased contrast sensitivity, confirmed by two independent tests. Only spatial frequencies higher than or equal to 3 cpd were enhanced.

Firstly, we confirmed our hypothesis that blue-light constricts the pupil more than red-light when illuminating the blind-spot meaning that the blind-spot is sensitive to blue-light. In a recent study [19], pupil response was measured following the same stimulus as in the blind-spot but additionally positioned in the parafovea and periphery which are stimulations of the visual field away of the blind-spot. A significant difference was found between parafovea and blind-spot condition for the time window 1s<1.7s, suggesting that missing rhodopsin in blind-spot leads to a lower response than in parafovea. For the time window showing exclusively the melanopsin mediated response i.e. 1s>1.8s, there was no significant difference between the blind-spot and the two conditions outside the blind-spot demonstrating a similar melanopsin mediated response under all three conditions. Furthermore, it was shown that equivalent stimulation in the blind-spot and periphery revealed comparable pupil responses for 1s>1.8s. Since eye movements were controlled by an eye tracker and none of the participants reported having perceived the stimulus in the blind-spot, this was attributed to activation of melanopsin in the axons of ipRGCs at the optic disc using blue-light [19]. Secondly, we revealed an improvement in contrast sensitivity following the stimulation of the blind-spot assessed by two reliable contrast sensitivity tests. This study showed for the first time an improvement in contrast sensitivity 20 min after blind-spot stimulation with blue-light. Our findings confirmed the hypothesis that contrast sensitivity was improved only at spatial frequencies higher than or equal to 3 cpd. The contrast sensitivity was not improved at lower spatial frequencies, suggesting the specificity of the blind-spot stimulation on spatial frequencies higher than or equal to 3 cpd. These results suggest that blind-spot stimulation modifies contrast sensitivity at particular spatial frequencies. Furthermore, the improvement of contrast sensitivity after blind-spot stimulation indicates an improvement on a subjective perceptual level.

In addition, we found the enhancement of the contrast after the blind-spot stimulation in two independent tests in which different monitors were used, however the ambient light was kept constant by carefully darkening the windows of the experimental room. The precision of stimulus presentation for the contrast sensitivity tests depends on the gray-level resolution and it is recommend to be at least 12.4 bits [25]. First with the FrACT we used a regular 8 bit monitor, whereas later we used the TueCST with a higher gray-level resolution monitor. The more accurate representation of the gray-levels could explain why the effect of increased contrast sensitivity 20 min after blind-spot stimulation is seen more clearly with the TueCST by stronger effect size (*η*_*p*_*^2^* = 0.218, *f* = 0.528 for FrACT vs *η*_*p*_*^2^* = 0.436, *f* = 0.88 for TueCST for blue light blind-spot stimulation main effect), although fewer participants were measured. The results support previous findings [17] that a monitor with higher gray levels is preferable for contrast sensitivity measurements.

Previous work of electroretinogram (ERG) measurements have revealed that blind-spot stimulation triggers retinal activity in the inner plexiform layer involving the dopamine system [7]. Of the different types of interneurons, amacrine cells modulate the output of ganglion cells [26]. On the retinal level, dopamine releasing amacrine cells receive retrograde signaling from melanopsin-containing ipRGCs [27,28], an interaction which may be reciprocal [29]. These findings imply a connection between melanopsin and dopamine. It has been shown that ipRGCs contribute to contrast sensitivity and that melanopsin deficient mice have reduced contrast sensitivity [30]. These results suggest that melanopsin plays a role in contrast sensitivity modulation, which the results of our study confirm, as contrast sensitivity was increased after blind-spot stimulation, which response is assumed to be driven by melanopsin although contributions from rods or cones cannot be completely excluded. An increase in dopamine has been shown to enhance contrast sensitivity for stimuli with spatial frequencies higher than 2 cpd in healthy participants [18]. We have observed a similar increase in contrast sensitivity in terms of spatial frequency pattern in our results after blind-spot stimulation on spatial frequencies higher than or equal to 3 cpd. This characteristic change in contrast sensitivity might be interpreted as an indirect proof of retinal dopamine change after stimulation of the blind-spot.

Although light falling on the blind-spot is not perceived consciously, it nevertheless contributes to perceptual processes, such as in the case of illusory contour interpolation [31]. Recent research has shown that illusory contours running through the blind-spot are represented similarly to an uninterrupted control [32]. Therefore, it could be that invisible stimulation of the blind-spot supports contour recognition at a cognitive level. It has also been reported that brightness perception of the light outside the blind-spot is modulated when the blind-spot is stimulated [2]. It appears that the blind-spot serves as a reference field for brightness perception calibration, which is likely facilitated by melanopsin [33].

Recently, a study investigating the effect of eye stimulation using different wavelengths showed no contrast sensitivity change after optic nerve head stimulation using blue-light [34]. In contrast to this study, we measured contrast sensitivity 20 min after stimulation and not immediately afterwards. ERG studies detected the increase in retinal activity only 20 min and not 10 min after the blind-spot stimulation [7,8]. It is possible that the contrast sensitivity improvement after blind-spot stimulation develops only after a certain time. Furthermore, we used VR-headset for the stimulation in our study and implemented more training trials.

A possible learning effect should be minimal or absent in our study, since participants were trained sufficiently with 10 training trials for each spatial frequency tested. Moreover, no improvement in contrast sensitivity below 3 cpd was obtained. With a learning effect, one would expect an improvement in contrast sensitivity across all spatial frequencies equally. Therefore, a learning effect can in our view not explain the full pattern of our results. In addition, a limitation of our study is the absence of a red-light control in experiment 2 (contrast sensitivity), whereas a red-light control was included in experiment 1 (pupil light response). Nevertheless, we had control conditions within the contrast sensitivity experiment by measuring spatial frequencies lower than 3 cpd. The control spatial frequencies were selected based on previous work [18]. There was no significant effect in the control conditions in our study. To exclusively state that the mechanism does not involve rods, future studies using a rod bleach or ERG should be considered. Even if the intensity of blue blind-spot stimulation is 13.8 log quanta.cm^-2^.s^-1^, which is above the threshold of melanopsin [21,22], it cannot be excluded that stimulation from other photoreceptors may superimpose the blind-spot stimulation. Another limitation is that eye movements could not be recorded in experiment 2. Nevertheless, careful instructions were given to the participants to maintain fixation on the fixation target throughout the 1 min blind spot stimulation. In experiment 1, eye movements were controlled by means of an eye tracker.

In conclusion, this study demonstrates that stimulating the blind-spot with blue-light constricts the pupil, presumably via melanopsin pathway, and increases the contrast sensitivity at higher spatial frequencies, suggesting an increase of retinal dopamine.

## Supporting information

S1 Data Spectrum of monitor FUJITSU Display B24-8 TS. (CSV)

S2 Data Spectrum of screen Samsung Galaxy S7. (CSV)

S3 Data Experiment 1 Pupil response 2s-6sAUC. (CSV)

S4 Data Experiment 1 Pupil response 6sPIPR. (CSV)

S5 Data Experiment 1 Pupil response 1.8sPIPR. (CSV)

S6 Data Experiment 1 Pupil response 1.7sPIPR. (CSV)

S7 Data Experiment 2 FrACT CS data. (CSV)

S8 Data Experiment 2 TueCSTest CS data. (CSV)
